# Age of Retirement and Human Capital in an Aging China, 2015–2050

**DOI:** 10.1007/s10680-018-9467-3

**Published:** 2018-02-13

**Authors:** Qiushi Feng, Wei-Jun Jean Yeung, Zhenglian Wang, Yi Zeng

**Affiliations:** 1 Department of Sociology, Centre for Family and Population Research (CFPR), National University of Singapore, Singapore, Singapore; 2 Department of Sociology, Centre for Family and Population Research (CFPR), Changing Family in Asia Cluster of Asia Research Institute (ARI), Faculty of Arts and Social Sciences, National University of Singapore, Singapore, Singapore; 3 Center for Population Health and Aging of Population Research Institute, Duke University, Durham, NC USA; 4 Center for the Study of Aging and Human Development, Duke University, Durham, NC USA; 5 Center for Healthy Aging and Development Study, Raissun Institute for Advanced Studies, National School of Development, Peking University, Beijing, China

**Keywords:** Retirement, Human capital, China, Aging, Projection

## Abstract

As China continues to age rapidly, whether the country should adjust the official retirement age, and if so, when and how, are currently major policy concerns. We examine the impact of postponing the retirement age on the human capital of China in the next four decades. Two critical aspects of human capital—health and education—are incorporated to account for the quality of the work force. Our projections reveal the impact of nine scenarios on the Chinese labor force in the next few decades, highlighting the changes in “the high human capital workforce”—those with good health and education. We show substantial impact with added work force ranging from 28 to 92 million per year depending on which scenarios are implemented. Furthermore, the retained workers are increasingly better educated. The gain in female workers is particularly significant, reaping the benefits of the education expansion since the 1990s.

**Electronic supplementary material:**

The online version of this article (10.1007/s10680-018-9467-3) contains supplementary material, which is available to authorized users.

## Introduction

Due to the joint effect of fertility decline and prolonged life span, population aging has become a global phenomenon, and the trend is expected to continue in the foreseeable future. In China, the world’s most populous nation, the proportion of population aged 65 and above is projected to triple from 9.6% in 2015 to 27.6% in 2050, and the old-age dependency ratio to increase from 0.13 to 0.47 during the same period (UNPD 2015a). In the face of such an unprecedented demographic transformation, policymakers are concerned about the shortage of working-age population, the affordability of the public assistance such as pension, the accessibility of the healthcare resources for elderly, and the sustainability of the family support system (UNFPA 2012; Harper 2014). This paper considers the impact of postponing retirement age on the size, stock, and quality of China’s workforce from 2015 to 2050.

Major policies developed in response to the aging trend across countries include, though not limited to, the following measures: (1) increasing the fertility rate, (2) adding young immigrants, (3) promoting productivity by enhancing human capital, and (4) extending the retirement age. Unfortunately, many policy protocols as currently proposed or adopted face substantial challenges. Currently, about 80 countries among the 186 countries in the world have fertility rates that are below the replacement level, namely 2.1 children per woman. Pronatal policies such as financial incentives, maternity or paternity leaves, and childcare provision have achieved limited success. Scholars have predicted that it is almost impossible for fertility in industrialized countries to restore to the replacement level in the near future (Kalwij 2010; United Nations 2012; Davies 2013). Policies that were designed to attract young foreign immigrants prove to be only a partial solution to offset the growth of the domestic elderly population, because the number of required immigrants would be “unrealistically large” (United Nations 2012:14). Furthermore, due to social and political complexities involved in international migration, most industrial countries are tightening controls over immigration nowadays (Hollifield et al. 2014). The positive association between education, productivity, and development has long been acknowledged (Havighurst 1953; Coleman 1965/2015; Psacharopoulos and Woodhall 1985). Education also positively relates to cognitive performance in old ages (see Albert 1995). More recently, enhancing human capital has been advocated as an effective option against the negative consequences of population aging (Lee and Mason 2010; Lutz and Samir 2011; Lutz 2014). However, promoting national educational system is a relatively slow process and of high cost. For low- and middle-income countries in particular, where 80% of elderly population will live in 2050, this is a gradual long-term solution (World Health Organization 2014).

China has started to lose its “first demographic dividend” in recent years due to an aging population (Mason 2005). Many national policies have been currently under heated debates regarding how best to make adjustments to counter the aging trend. For example, the Chinese government has begun to relax its one-child policy, first allowing couples where at least one partner is a single child to have two children in 2014, and then most recently ended the one-child policy officially to allow all couples to have two children. These adjustments have thus far had limited impact (the Economist 2015; Zhai and Li 2015). To what extent this policy change will alleviate challenges due to population aging in China remains to be seen. While the total fertility rate is generally expected to increase gradually in a limited scope in the near future, it will be at least two decades before this policy change has a notable impact on the share of the working-age population in China. Apart from the relaxation of one-child policy, the Chinese government has created a national pension system, in particular expanding coverage to the long neglected rural population, which is projected to reach a full coverage by 2020 (Chen 2014). However, the sustainability of this national pension system has been seriously questioned under the rapid population aging (Oksanen 2010; Zeng 2011).

With population aging, the shortage of labor supply is expected to increase wage and hurt the nation’s global competitiveness, hence increase the risk for China to fall into the “middle-income trap.” As a counter measure, China has substantially expanded its education enrollment since the 1990s, increasing the college enrollment by sevenfolds since 1999, in order to improve the quality of its labor force. The increase has been particularly fast for females, with the female gross tertiary education enrollment surpassed that for males since 2009 (Yeung 2013). The national expenditure on human resources has also increased significantly at the same time. Despite the big push to 1 in 4 Chinese youth aged 18–21 enrolled in colleges now, the level of higher education in China remains much lower compared to OECD countries (World Bank 2015).

### Raising Retirement Age as a Solution to the Rapid Aging Trend

Postponing age of retirement is currently another key potential policy solution for enhancing human capital under discussion in China. Since the 1950s, China has implemented a compulsory scheme to regulate retirement age, with 60 for men, 55 for female professionals/cadres (including teachers, medical personnel, other professionals, and administrators), and 50 for the rest of the female workers. To this day, this scheme has had no substantial changes despite the dramatic socioeconomic transformations in the last three decades. Postponing the official retirement age, however, is a thorny political initiative, facing high resistance especially from those who are approaching retirement. According to a recent survey conducted by Manulife (2014), a financial company, about 64% of interviewees from China had a negative attitude toward such a policy change.

Many industrialized countries have had mandatory retirement ages and have extended the age of retirement in recent years (Hardy 2011). Table 1 lists the official retirement age (i.e., the legally designated age for pension), effective retirement age (i.e., actual age to withdraw from labor market), and proposed future adjustment on pension age among selected OECD countries. By 2050, the official retirement age can be expected to be postponed to at least 67 in most OECD countries (OECD 2013). Some OECD countries even link pension ages to changes in life expectancy, suggesting a further delay in retirement age beyond 67. Moreover, as listed in Table 1, although the gender gap in retirement age, namely women retire earlier than men, is currently quite common among OECD countries, it is expected to be eliminated in the near future, according to these policy proposals.

**Table 1:** Official age of retirement, average effective age of retirement, and proposed adjustments of retirement age in selected OECD countries. *Source*: based on OECD (2013), and related government websites

Country	Official age of retirement		Effective age of retirement		Proposed adjustments of retirement age		
	Men	Women	Men	Women	Target age of adjustment	Ending year of adjustment	Gender specificity in adjustment
Australia	65	65	64.9	62.9	70	2035	N
Austria	65	60	61.9	59.4	65	2033	N
Belgium	65	65	59.6	58.7	67	2030	N
Canada	65	65	63.8	62.5	67	2029	N
Czech Republic	62.5	61.3	63.1	59.8	66.7	2019/2030	N
Denmark	65	65	63.4	61.9	67	2019/2022	N
Estonia	63	61	63.6	62.6	65	2026	N
Germany	65	65	62.1	61.6	67	2031	N
Greece	65	65	61.9	60.3	67	2025	N
Hungary	63.5	63.5	60.9	59.6	65	2022	N
Ireland	66	66	64.6	62.6	68	2028	N
Israel	67	62	66.9	65.1	64	NA	Female only
Italy	66	62	61.1	60.5	67	2021	N
Korea	60	60	71.1	69.8	65	2033	N
Netherlands	65	65	63.6	62.3	67	2021	N
Poland	65	60	62.3	60.2	67	2020/2040	N
Slovak Republic	62	59.8	60.9	58.7	62	NA	Female only
Slovenia	63	61	62.9	60.6	65	2016	N
Spain	65	65	62.3	63.2	67	2027	N
Turkey	60	58	62.8	63.6	65	NA	N
USA	66	66	65	65	67	2022	N
UK	65	61.2	63.7	63.2	66	2018/2022	N
OECD average	64.7	64.2	63.4	63.1			

The official age is the age at which a pension can be received irrespective of whether a worker has a long insurance record of years of contributions. And the average effective age of retirement is defined as the average age of exit from the labor force during a 5-year period

The rationale behind these adjustments on retirement age is that the old pension programs penalize work in old age and encourage early retirement when population aging makes it necessary to prolong the working lives to compensate for the increasing public eldercare costs (Gruber and Wise 2007). The consequent unsustainability of pension system and shortage of labor supply are thus driving more than half of the governments in the world to change their retirement systems (UNPD 2015b). A prolonged retirement age as a major measure in these reforms arguably has “a triple dividend,” namely to boost labor force, to improve public finance, and to smooth the pace for employers to replace retiring workers (OECD 2006:24). There are indeed other related policy measures such as cutting pension benefit or strengthening penalty of early retirement (OECD 2006). However, all these measures are controversial politically, especially given that old-age pension has become a moral commitment in many welfare states. Politicians thus often have to make the reform schemes complicated to “obfuscate” non-experts, to use long phasing-in periods to reduce oppositions, or to develop occupation-specific policy changes to divide potential opponents (Kohli and Arza 2011).

Extending the retirement age has important implications for the Chinese social and economic prospects. To delay the retirement age could directly enhance the sustainability of the national pension account, with more contributing working-age individuals and less payout-receiving retirees. In addition, doing so will also increase the size and stock of the labor force in China, which is pivotal for the future productivity and economic growth. In particular, due to the educational expansion noted above, postponing the age of retirement can be expected to not only retain more people in the labor force but retain those better educated than their predecessors. Because of the larger increase in education among females relative to males in the last few decades and the current lower retirement age for females, the gain for better-educated women in the next three to four decades will be particularly large.

To raise retirement age effectively, however, depends on a thorough understanding of the levels, trends and determinants of age-specific productivity potential. Several studies (McEvoy and Cascio 1989; Skirbekk 2008), for example, conclude that on average the age–productivity curve tends to be inverse u-shaped, wherein productivity lowers when health (for example, disability) and cognitive skills (such as memory and processing speed) decline toward the end of the working life. In the current policy discussions on retirement age in China, it is necessary to examine how various scenarios of the retirement age adjustment affect the size, stock, and quality of the national human capital. To our knowledge, there has been little investigation on these issues.

In this paper, we consider the major current policy proposals, propose nine schemes of retirement age adjustments in the next few decades, and project the size, stock, and quality of China’s workforce from 2015 to 2050. To address “quality” of the human capital, we incorporate health and education in the calculation. We then compare the extent to which each adjustments scenario affects the compositions of the labor market and support ratios, and discuss their policy implications. Results show very substantial impact with added work force ranging from 28 to 92 million per year. In the following sections, we present nine different potential adjustment schemes. Then we introduce the projection method, present major findings, and finally conclude and discuss policy implications.

## Potential Schemes of Retirement Age Adjustment

Public responses toward changes in official retirement age tend to vary by factors such as when the change starts, for how long, the scope of change, and to whom the changes apply. We present the various schemes proposed in China in recent years in Table 2. These policy proposals differ in the targeted age of retirement, when to begin the adjustment, span of changes, and gender-specific schedules. With these past proposals as references, we develop nine schemes of retirement age adjustment based on the key parameters as below.

**Table 2:** Major policy proposals for retirement age adjustment in China

Proposals	Target age of adjustment	Time of adjustment	Gender specificity in adjustment	Details
Lin (2001)	65	2001–2045	N	Females reach a retirement age of 55 by 2015, and then reach 60 by 2030, and both males and females reach 65 by 2045
Liu and Miao (2004)	65 for males and 60 for females	2004–2050	Y	Both males and females postpone 1 year in retirement per 5 years for 2021–2040, and then postpone 1 year for 2041–2050
Sun (2005)	65 for male and female cadres, and 60 for female workers	2005–2015	Y	Both males and females postpone retirement by 1 year annually
Shao and Nie (2007)	60	2010–2050	N	Female workers delay 1 year in retirement per 2 years for 2010–2020, all females delay 1 year per 2 years for 2020–2030, and both females and males delay 1 year per 3 years for 2030–2045
Yang (2013)	65	2015–2030	N	For males, pension is delayed for 6 months after retirement, and for females, 12 months
Ministry of Human Resources and Social Security (2013)	65	2010–2030	N	Change of female starts from 2010, and change of male starts from 2015
Wang (2014)	65	2015–2050	N	Change of female starts from 2015, and change of male starts from 2030
Chinese Academy of Social Sciences (2014)	65	2018–2045	N	For females, retirement is delayed for 1 year per 3 years, and for males, 1 year per 6 years

### Adjustment Parameters

Based on the review of current policy proposals, we use the following parameters to construct schemes of retirement age adjustment.

#### Targeted Age of Retirement

We propose three options for adjusting age of retirement for men and women: (1) increase by 5 years for each of the three groups (i.e., to 65 years for men, 60 for female professionals/cadres, and 55 for the rest of female workers), (2) 60 for all females and 65 for all males, and (3) 65 for both females and males. From recent statistics, we see that the proportion of those aged 60–64 who are not able to carry out regular work and daily activities are low—3.4% for females and 4.3% for female in 2005 (see Table 8 of Appendix). Hence, we think postponing age at retirement up to 65 is feasible.

#### Gender-Specific Schedule

If the targeted retirement age is 65 for both females and males, we consider two options to adjust for men and women: (1) first increase female’s retirement age to male’s current age at retirement (60) and then increase the age together for both genders to 65, or (2) starting at the same time to increase the age of retirement for male and female to 65, with a faster rate of annual increase for females (since females start at 50 or 55 and males start at 60).

#### Beginning Time

We set two different time points to start the adjustment: (1) start immediately (as of 2015),^1^ and (2) start 10 years later (i.e., in 2025). A late start will affect only future cohorts and thus may meet a lower public resistance now; however, there is a trade-off in that the later cohorts will need to face a steeper increase to achieve the same targeted size of labor force.

**Fig. 1:**
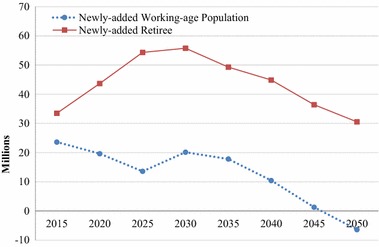
Newly added working-age population and retirees by every 5 years in urban China if current retirement ages remain unchanged, 2015–2050

The policy will likely face less political resistance if the retirement age is raised at a time when the employment pressure is decreasing (see Lin 2001). In Fig. 1,^2^ we project the number of newly added working-age population and retirees in urban China for each 5-year age group from 2010 to 2050, assuming there is no change to the current retirement ages. It can be seen that the two beginning time of our adjustments, 2015 and 2025, appear to be feasible choices for postponing retirement age, as the newly added working-age population is generally declining after these 2 years, although there is a short period of relatively small increase from 2025 to 2030. As noted earlier, considering the need to offset the growth of newly added retirees, the beginning time set as 2015 could be even better than 2025 as we can see from Fig. 1 that retirees increase quite rapidly from 2015 to 2030.

#### Span of Change

We propose to use 25 years as the span of change in the retirement age adjustment. This duration will generate a gradual annual change. For example, for males changing from age 60 to 65, a span of 25 years means a delay of about 2 months per year, and for females from age 55 to 65, a delay of 5 months per year. Of course, a longer duration could lead to an even smaller annual change, yet it also implies a longer period of time is needed to achieve the target age of retirement.

### Adjustment Schemes

Based on the combination of these parameters, we propose the following nine schemes, which are used to derive scenarios of changes in the size, stock, and quality of China’s labor force in the next few decades. We summarize these scenarios in Table 3. We denote the two sets of schemes for *early (2015)* and *late (2025)* starting time with a suffix of *e* and *l*, respectively.

**Table 3:** Nine schemes of retirement age adjustment in China

Scheme	Target retirement age- and gender-specific schedule	Beginning time and span of change		
		2015–2050	2015–2040 (Early scheme)	2025–2050 (Late scheme)
Scheme A	The current official ages remain unchanged: 50 for female workers, 55 for female cadres, and 60 for all males	No change (Scheme A)	–	–
Scheme B	Everyone prolongs retirement by 5 years: 55 for female workers, 60 for female cadres, and 65 for all males	–	Add 5 years early (Scheme B_e)	Add 5 years late (Scheme B_l)
Scheme C	All females retire at the same age: 60 for all females and 65 for all males	–	Female 60–male 65–early (Scheme C_e)	Female 60–male 65–late (Scheme C_l)
Scheme D	Everyone retires at 65: 65 for all, with adjustment for both genders at the same time	–	65 for all–early (Scheme D1_e)	65 for all–late (Scheme D1_l)
	Everyone retires at 65 but females adjusted first: 65 for all, with adjustment for females to 60 first and then for both genders to 65	–	65 for all–female first–early (Scheme D2_e)	65 for all–female first–late (Scheme D2_l)

- *No Change (Scheme A)* This is a baseline scheme as the reference for comparison, in which the current retirement ages remain unchanged from 2015 to 2050, namely 60 years for men, 55 for female professionals/cadres, and 50 for other female workers.
- *Add 5 Years (Scheme B_e and Scheme B_l)* These two schemes add 5 years to the current retirement age for each of the three groups—male, female professional/cadre, and female workers, respectively. That is, the retirement age will gradually be postponed from 50 to 55 for female workers, from 55 to 60 for female professionals/cadres, and from 60 to 65 for males, within the 25-year period of change. The only difference between these two schemes is that Scheme B_e starts in 2015, while B_l starts in 2025. In reality, the Chinese government has already initiated trials that allow female professionals/cadres to retire at the age of 60.
- *Females 60, Males 65 (Scheme C_e and C_l)* These two schemes eliminate the gap between female workers and female professionals/cadres. That is, the retirement age will be gradually postponed from 50 to 60 for female workers, from 55 to 60 for female professionals/cadres, and from 60 to 65 for males, within the 25-year period. Scheme C_e will start in 2015 and C_l will start in 2025.
- *65 for All (Scheme D1_e and D1_l)* These two schemes further eliminate the difference in age of retirement between females and males. That is, both men and women will gradually postpone the retirement age to 65 within the 25-year period. The rate of change will be faster for females than for males. Adjustments for each of the three groups will begin at the same time but with different rates of adjustment. Scheme D1_e will start in 2015 and D1_l will start in 2025.
- *Female First, 65 for All (Scheme D2_e and D2_l)* These two schemes are almost the same as Scheme D1_e and Scheme D1_l, except that the female’s age of retirement will be postponed first to reach male’s level at 60 in the first 15 years, and then both genders will increase together from 60 to 65 in the next 10 years.

These alternative scenarios are expected to affect the labor force in different degrees and generate different public responses. There are trade-offs for adopting each scheme. Clearly, setting an oldest possible age, starting postponing the age of retirement as soon as possible, and reaching the target as quickly as possible will definitely generate most gains in the labor force. However, these proposals are also more likely to incite more resistance from the public, especially from those who will be reaching the current retirement age soon.

## Method

### Population Projection by ProFamy

We use the most recent 2010 census of China as the baseline to project the age–sex–rural/urban-specific distribution of population in China to 2050. The projection is done by the ProFamy Extended Cohort-component Method (thereafter ProFamy). This method is developed by Zeng et al. (1997, 1998), further extended by Zeng et al. (2006, 2013), and have been employed in various demographic projections (e.g., Feng et al. 2011). ProFamy uses an individual-based macro-simulation to forecast population and number of household; it is essentially a cohort-component method, in which projections of changes in demographic components (including fertility, mortality, marriage/union, co-residence of children/parents, and migration) are made for each of the cohorts that produce population and household distributions in future years. Validation tests from 1990 to 2000 using the ProFamy model and based on the observed US and Chinese demographic rates before 1991 show that forecast errors measured by discrepancies between the ProFamy projected values and the census observations are reasonably small (Zeng et al. 2006, 2008).

Although ProFamy has been used to project future household trends, it is also a valid approach for forecasting population. In projecting the future Chinese population, different from the other population projections such as the World Population Prospects by the United Nations and the international program of the US census Bureau, the ProFamy projection uses a rural–urban-specific model. That is, the ProFamy method projects by rural and urban regions separately with different standard schedules and parameters specific to these regions, which provide more precise estimations (Zeng and Vaupal 1989; Shen and Spence 1996). In China where there are large disparities between urban and rural development, this method is particularly useful. Moreover, the ProFamy method applies the fertility occurrence/exposure rates by parity estimated from micro-level census data, instead of fertility frequencies that are commonly adopted in the other projections. Lastly, the ProFamy projection is based on a simulation of individual’s marital status and the number of children ever born and thus could give more accurate estimates of future new births.

We restrict our projections to the urban areas of China. There are two systems of retirement in China, a formal one for urban employees with pension entitlement, and an informal system for the rural residents and urban unemployed individuals who do not have pension and have to rely on family support and prolong their working lives (Giles et al. 2012). In the absence of pension, most rural elderly work until as long as they could in a model of “ceaseless toil” (Benjamin et al. 2003), and the decisions to continue work in old age was mainly affected by the income and health of the elderly (Cai et al. 2012). The National Rural Pension Scheme (NRPS) was recently introduced in 2009. However, this new initiative is not expected to change the model of “ceaseless toil” in a short term. Under NRPS, rural participants pay a premium of 100–500 yuan per year for at least 15 years, and by age 60, receive a basic pension of 55 yuan plus 1/139 of the accumulated premiums per month. As noted by scholars, this benefit level is too low to facilitate rural elders to retire (Shen and Williamson 2010; Dorfman et al. 2013) as the basic pension is only about 15% of the average monthly expense of the Chinese rural resident in 2010 (about 365 yuan). In this sense, the current retirement policy for rural elderly has different implications from their urban counterpart. That is, the retirement age adjustment as proposed in this analysis is not applicable to the rural residents, and the policy priority for the rural retirement reform may instead focus on raising the benefit level. This also suggests that there is a long way ahead for China to achieve a universal social security system.

### Major Assumptions on Demographic Parameters

In these projections, we make assumptions based on extensive support from extant literature about the major demographic trends in China in the next few decades. For the fertility trend, various scholars estimated that the total fertility rate (TFR) in China ranged from approximately 1.5–1.8 since the end of the 1990s (e.g., Zhang and Zhao 2006; Zeng 2007; Zhao and Zhang 2010). Based on the 2010 and 2000 census data, demographic analysis of “forward forecasting” and “backward forecasting,” and adjustment for the under-reporting of births and children, we estimate that TFR in China in 2010 was 1.63. This estimate is supported by a recent study that uses the newly released national household registration data in China and concludes that the TFR of China should be at least 1.63 in the year of 2010 (Zhai et al. 2015). Based on the rural–urban TFR differences observed in censuses, we further estimated that the period TFRs in 2010 were 2.01 for rural areas and 1.24 for urban areas in China. Considering the recent revocation of the national one-child policy, we expect that TFR will increase from 1.24 in 2010 to 1.72 in 2050 for urban, and from 2.01 to 2.13 for rural areas.

Regarding the life expectancy, we assume a gradual improvement in mortality in China during the period of 2010–2050 from a life expectancy of 74 years for both sexes combined in 2010, to 81.8 years in 2050, and the gender differentials in life expectancy remain the same as that observed in 2010. Under this rationale, we assume the life expectancy at birth increases from 77.87 to 83.99 for urban females, from 74.21 to 80.54 for urban males, from 73.88 to 80.72 for rural females, and from 70.07 to 76.50 for rural males. These parameters are comparable to those in the World Population Prospects by the United Nations (UNPD 2015a).

As for the future urbanization trend, we assume that the urban population in China will increase from 50.27% in 2010 to 70.94%, and 86.95% in 2030 and 2050, respectively. These parameters are consistent with Chinese government’s goal of reaching 70% urbanization by 2030 stated in its 13th five-year plan. They are relatively higher than those assumed by World Urbanization Prospects (UNPD 2014) and projections of the World Bank (2014). In the World Urbanization Prospects, for instance, China’s urbanization rate in 2050 is set as 75.8%, lower than the level of the developed countries (85.4%). But urbanization rates in this projection match with the S shape of the logistic model as applied in the urbanization projections of UNPD and World Bank, namely; it will accelerate first then decelerate and followed by a plateau. Urbanization has accelerated rapidly in China in the past three decades with the annual increase in urban population at 0.70, 0.98, and 1.37 percentage points in the periods of 1980–1990, 1990–2000, and 2000–2010, respectively. According to the international experience, when urbanization reaches above 50%, the scale of change becomes smaller. In our projection, we assume the urbanization rate in China will decelerate with the annual rates of 1.05 and 0.80 percentage points in the periods of 2010–2030 and 2030–2050, respectively.

Moreover, these urbanization parameters may better capture the mechanism and progress of Chinese urbanization. Unlike the western experience, the rapid urbanization in China since the economic reform in the late 1970s has been greatly driven by the government. Especially in recent years, urbanization has been considered by Chinese policymakers as a major engine of the future economic growth. In the recent 18th National Congress of the Chinese Communist Party held in 2012, for example, urbanization has been strongly prioritized in the national economic plans. Due to these new initiatives, we believe our assumptions in urbanization parameters are justifiable. In addition, these parameters are also supported by recent literatures. For example, according to Gao and Wei (2013), using the curve-fitting method, the urbanization rate in China will reach 84.97% in 2050, and using GDP to predict urbanization in a log-linear model, the result is similarly at 84.83%. Both estimates are close to our assumed rates. More details about the assumptions and parameters used in our projections are summarized in Table 7 of Appendix.

### Assumptions on Distribution of Education and Health

We use health and education as the two primary aspects of human capital to assess the quality of labor force. We adopt a series of assumptions about the future age–sex-specific distributions of education and health based on micro-level census data, in order to model the future changes in the quality of labor force. The parameters of health and education are applied after rural/urban–sex–age-specific distribution of population was generated through the ProFamy simulation.

With regard to health, we use the data of National 1% Population Survey in 2005 to acquire the age–gender-specific prevalence of health status in 2005. The sample contains a measure that asks whether one is healthy to work and live on a regular basis. We use a crude indicator of health, classifying an individual as “disabled” if he or she self-reported to be “unable to carry out regular work and daily activities,” or to be “not sure about their health status.” Those who reported “not sure” (less than 2% of the population and the majority of them are older than 70) are also included in the unhealthy category. The “healthy” category includes those who self-reported as “healthy” or as “basically can carry out regular work and daily activities.”

There are two competing hypotheses in extant literature about the health trends under population aging. One group of scholars argues that due to the advances in medical technology, improvements in lifestyle and socioeconomic development, morbidity among the elderly may be “compressed” (Fries 1980). In contrast, another group of scholars believes that the reduced mortality may result in more frail elderly surviving with health problems, thus worsening the overall health of the elderly population (Gruenberg 1977). In reality, these two mechanisms may coexist and interact (Manton 1982; Robin and Michel 2004). That is, the health trend may change direction due to interactions of these two mechanisms. As literature suggests that the direction of the disability trends in China is not clear for the recent decades (see Du and Wu 2006; Gu and Zeng 2006; Feng et al. 2013), we assume that the distribution of health will maintain at a stable level from 2010 to 2050. The assumptions on the future distribution of health status are summarized as the medium scenario in Table 8 of Appendix. Due to the uncertain health trends, we also conducted robustness analysis with two additional scenarios, one with an increasing trend of unhealthy conditions (the high disability scenario) and the other with a decreasing trend of unhealthy conditions (the low disability scenario). For the high scenario, we assume the proportion of unhealthy Chinese elderly (50 +) to gradually increase by 10% from 2005 to 2050; for the low scenario, we assume the proportion of unhealthy Chinese elderly (50 +) decrease by 10% from 2005 to 2050. The results of these two scenarios are similar to those with the medium scenario, thus we do not present them in the main text, but show them in Fig. 8 in Appendix.

For education, we use the 1% sample data of the 2010 China Census for the age–sex-specific distribution of education in 2010, which is measured by five levels, ‘no schooling,” “primary school,” “junior middle school,” “senior middle school,” and “college and above.” For the future trends of education, we firstly assume that the college education ratio of cohorts aged from 20 to 29 will gradually increase to 58% by the year of 2050 in urban China for both genders. Such targeted prevalence in the tertiary education enrollment is similar to the levels for OECD countries in the early 2000s (World Bank 2015) and also matched the expectation in the literatures (see Yeung 2013). Then we could determine the distribution of education for the two age groups—20–24 and 25–29, at every 5-year interval from 2010 to 2050. For the future education distribution of other age groups older than the age of 30, we derive from the education distributions of the earlier corresponding cohorts. For example, education distribution of those aged 30–34 in 2020 is the same as the education distribution of those aged 25–29 in 2015. The details of the assumptions on the future distribution of education are summarized at Table 9 of Appendix.

Based on the combination of education and health status, we divide the entire population aged 15 and above into the following five groups: (1) unhealthy and thus not able to participate in the labor force, (2) healthy but retired, (3) healthy, not retired, and without any formal schooling, (4) healthy, not retired, and with an education of primary school or junior middle school, and (5) healthy, not retired, and with education of high school and above. To characterize those with higher human capital, we name the last group as *the high human capital workforce,* abbreviated as *high HC workforce* thereafter.

## Results

**Fig. 2:**
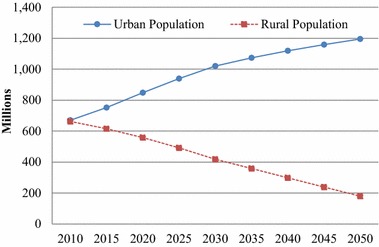
Rural and urban population in China, 2010–2050

Based on our projections with assumptions described above, the total population of China will grow from 1.33 billion in 2010 to 1.45 billion in 2030, and then gradually decline to 1.40 billion by 2050; and the urban population will increase substantially from about 670 million in 2010 to 1208 million in 2050 (see Fig. 2). Of the urban population, the working-age population, i.e., those aged 15 to 64, will increase from 497 million in 2010 to about 709 million in 2030, and then remain relatively stable at around 684 million until 2050. Such a trend may seem inconsistent with some other projections such as World Population Prospects by the UNDP and projections of International Institute for Applied Systems Analysis (IIASA), which predict that the working-age population in China may decline soon. However, we remind the readers that our projected trend is for the urban working-age population only, and the rapid urbanization in China which brings in large volumes of working-age population may partly explain the growth of this subpopulation. Moreover, as discussed above, our estimates of TFR are relatively higher than those of UNPD and IIASA.

**Fig. 3:**
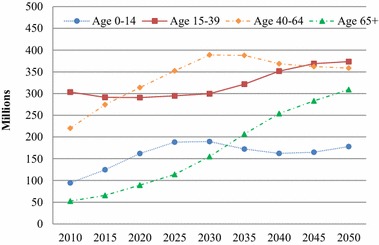
Urban population by four age groups in China, 2010–2050

We further divide the working-age population into two subgroups for age 15–39 and age 40–64 (Fig. 3). It is interesting to observe that these two subgroups have different trajectories of change from 2010 to 2050. The younger group, age 15–39, is stable at about 300 million until 2030 and then increase to about 370 million in 2050; in contrast, the older group, age 40–64, first doubled from about 200 million to 400 million from 2010 to 2030, and then gradually decline to about 350 million in 2050.

### Size of Work Force

We first show the relative size of work force and retirees from 2010 to 2050 under each of the nine scenarios in Table 4. As shown, although the total urban population in China will continue to increase from 2010 to 2050, if the current retirement ages remain unchanged by 2050 (Scheme A), the total workforce will only increase by 17% in 2030 and by 22% in 2050, and the number of retirees will increase much more rapidly by about 1.5 times in 2030 and about 3 times in 2050. In contrast, delaying age of retirement under Schemes B (*Add 5 Years*), C (*Females 60, Males 65*), and D (*65 for All*) could change these trends in various degrees. For example, Scheme D will yield the largest gain in the size of the work force, generating a 55% increase in the workforce, and only a 1.7 times increase in retirees in 2050. This scheme of adjustments will generate the most substantial increase for women, with female workforce expected to increase by 68% and female retirees increase only by 118% (as compared to 17 and 256%, respectively, in the *No Change* scenario under scheme A). We also see a strong impact, though with a smaller magnitude, on the trend for male workforce. The other schemes generate impacts that fall in between Schemes A and D.

**Table 4:** Relative change of the size of workforce and retiree in China under nine retirement schemes with the 2010 population as baseline, for the years of 2030, 2040 and 2050

	Total		Female		Male	
	Workforce	Retiree	Workforce	Retiree	Workforce	Retiree
Baseline population in 2010	460,967,916	114,727,989	207,105,294	76,545,247	253,862,622	38,182,741
*Relative change in 2030 under nine retirement schemes*						
A	17%	163%	11%	147%	22%	196%
B_e	26%	125%	20%	120%	31%	134%
B_l	20%	150%	14%	138%	25%	176%
C_e	30%	110%	29%	98%	31%	134%
C_l	21%	146%	16%	131%	25%	176%
D1_e	35%	89%	40%	66%	31%	134%
D1_l	22%	140%	20%	122%	25%	176%
D2_e	31%	104%	43%	58%	22%	196%
D2_l	21%	146%	20%	121%	22%	196%
*Relative change in 2040 under nine retirement schemes*						
A	23%	245%	16%	215%	28%	307%
B_e	40%	177%	37%	158%	42%	214%
B_l	33%	204%	29%	181%	37%	251%
C_e	46%	154%	50%	125%	42%	214%
C_l	37%	189%	37%	158%	37%	251%
D1_e	54%	122%	67%	76%	42%	214%
D1_l	42%	169%	48%	129%	37%	251%
D2_e	54%	122%	67%	76%	42%	214%
D2_l	38%	185%	50%	125%	28%	307%
*Relative change in 2050 under nine retirement schemes*						
A	22%	304%	17%	256%	26%	400%
B_e	39%	235%	32%	215%	44%	275%
B_l	39%	235%	32%	215%	44%	275%
C_e	44%	214%	43%	184%	44%	275%
C_l	44%	214%	43%	184%	44%	275%
D1_e	55%	170%	68%	118%	44%	275%
D1_l	55%	170%	68%	118%	44%	275%
D2_e	55%	170%	68%	118%	44%	275%
D2_l	55%	170%	68%	118%	44%	275%

The nine retirement schemes are A (the current retirement ages remain unchanged), B_e (everyone will prolong retirement by 5 years from 2015 to 2040), B_l (everyone will prolong retirement by 5 years from 2025 to 2050), C_e (females will retire at 60 and male at 65 from 2015 to 2040), C_l (females will retire at 60 and male at 65 from 2025 to 2050), D1_e (everyone will retire at 65 from 2015 to 2040), D1_l (everyone will retire at 65 from 2025 to 2050), D2_e (everyone will retire at 65 from 2015 to 2040 with females adjusted first), D2_l (everyone will retire at 65 from 2025 to 2050 with females adjusted first)

**Fig. 4:**
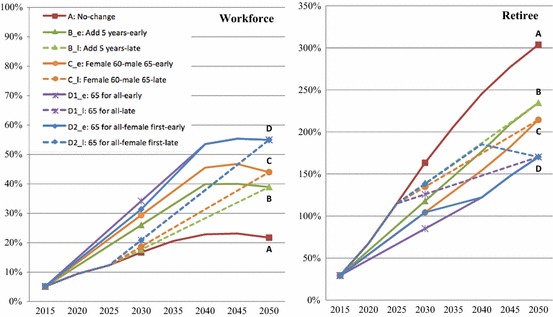
Relative changes of the size of workforce and retiree under nine retirement schemes with the 2010 population as baseline, 2015–2050. The nine retirement schemes are A (the current retirement ages remain unchanged), B_e (everyone will prolong retirement by 5 years from 2015 to 2040), B_l (everyone will prolong retirement by 5 years from 2025 to 2050), C_e (females will retire at 60 and male at 65 from 2015 to 2040), C_l (females will retire at 60 and male at 65 from 2025 to 2050), D1_e (everyone will retire at 65 from 2015 to 2040), D1_l (everyone will retire at 65 from 2025 to 2050), D2_e (everyone will retire at 65 from 2015 to 2040 with females adjusted first), D2_l (everyone will retire at 65 from 2025 to 2050 with females adjusted first). D1_l and D2_l are presented as one at the left panel due to the minor difference

We present these patterns graphically in Fig. 4 to further illuminate the expected impact over time on the workforce and retirees under different schemes. It is evident that the beginning time matters, with schemes that begin earlier generating a substantially larger and quicker impact than those that start 10 years later. For example, schemes that start in 2015 all will generate a larger increase in the size of the workforce by 2030, compared to their corresponding schemes that start in 2025. Under Scheme B_e (*Add 5 Years*, starting from 2015), the size of the workforce will be 581 million by 2030, an increase of 26% (linear estimation) from 2010, whereas under Scheme B_l (*Add 5 Years*, starting from 2025), the workforce size will be 542 million in the same year, amounting to an increase of 20%.

Moreover, whether the retirement age began to be adjusted at the same time for men and women also generates different impact. There are notable differences between Schemes D1_e (*65 for All,* starting from 2015) and D2_e (*Female First, 65 for All, starting from 2015*), and between Schemes D1_l (*65 for All,* starting from 2025) and D2_l (*Female First, 65 for All,* starting from 2025), with regard to changes in the number of retirees. For instance, under Scheme D1_l, the total retirees in 2040 will be 309 million, an increase of 169% from 2010, but if Scheme D2_l is adopted instead, the size will increase to 327 million (an increase of 185%).

### Quality of Work Force

**Fig. 5:**
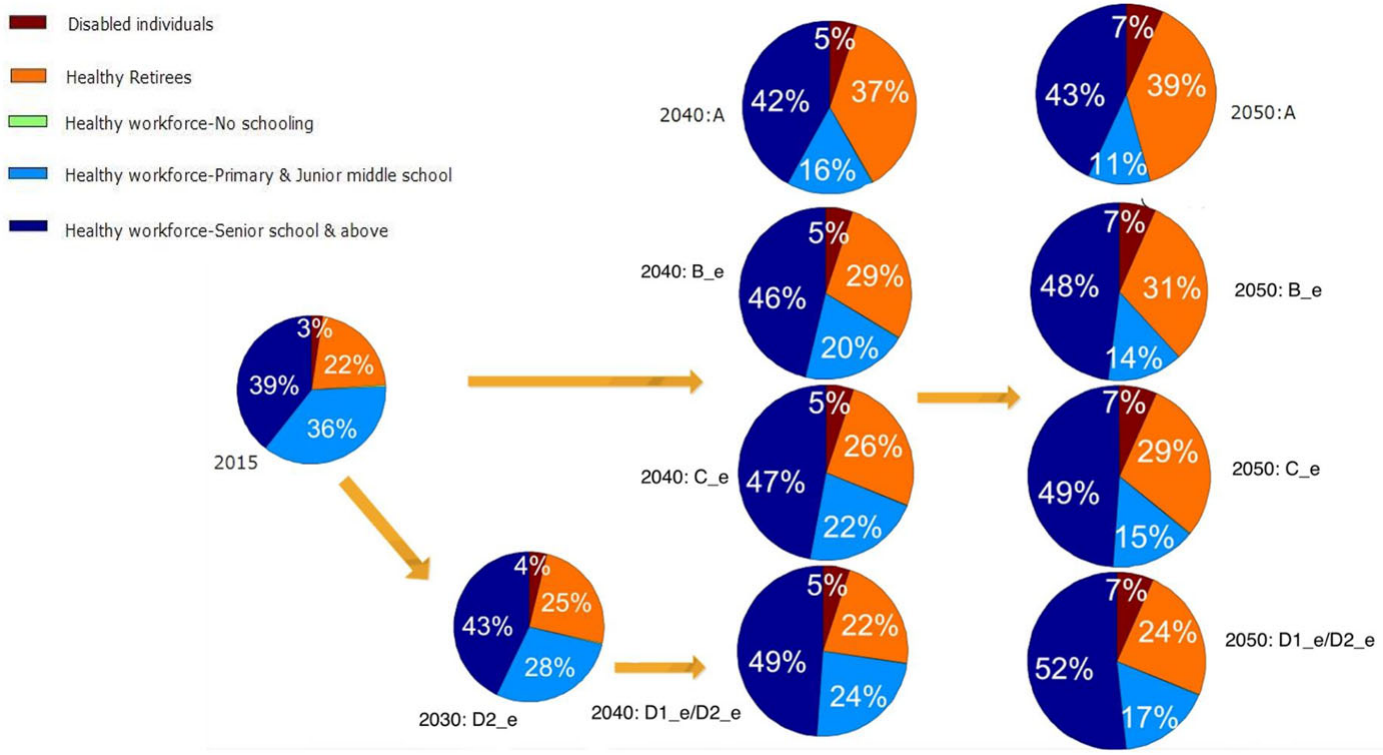
Working-age population in China by education and health under early retirement schemes adjusted from 2015 to 2040. The early retirement schemes include A (the current retirement ages remain unchanged) as the reference scheme, B_e (everyone will prolong retirement by 5 years from 2015 to 2040), C_e (females will retire at 60 and male at 65 from 2015 to 2040), D1_e (everyone will retire at 65 from 2015 to 2040), D2_e (everyone will retire at 65 from 2015 to 2040 with females adjusted first). An individual is categorized as “disabled” if he or she self-reported to be “unable to carry out regular work and daily activities,” or to be “not sure about their health status.” Those who reported “not sure” were less than 2% and the majority of them are older than age 70. The “healthy” category includes those who self-reported as “healthy” or as “basically can carry out regular work and daily activities”

**Fig. 6:**
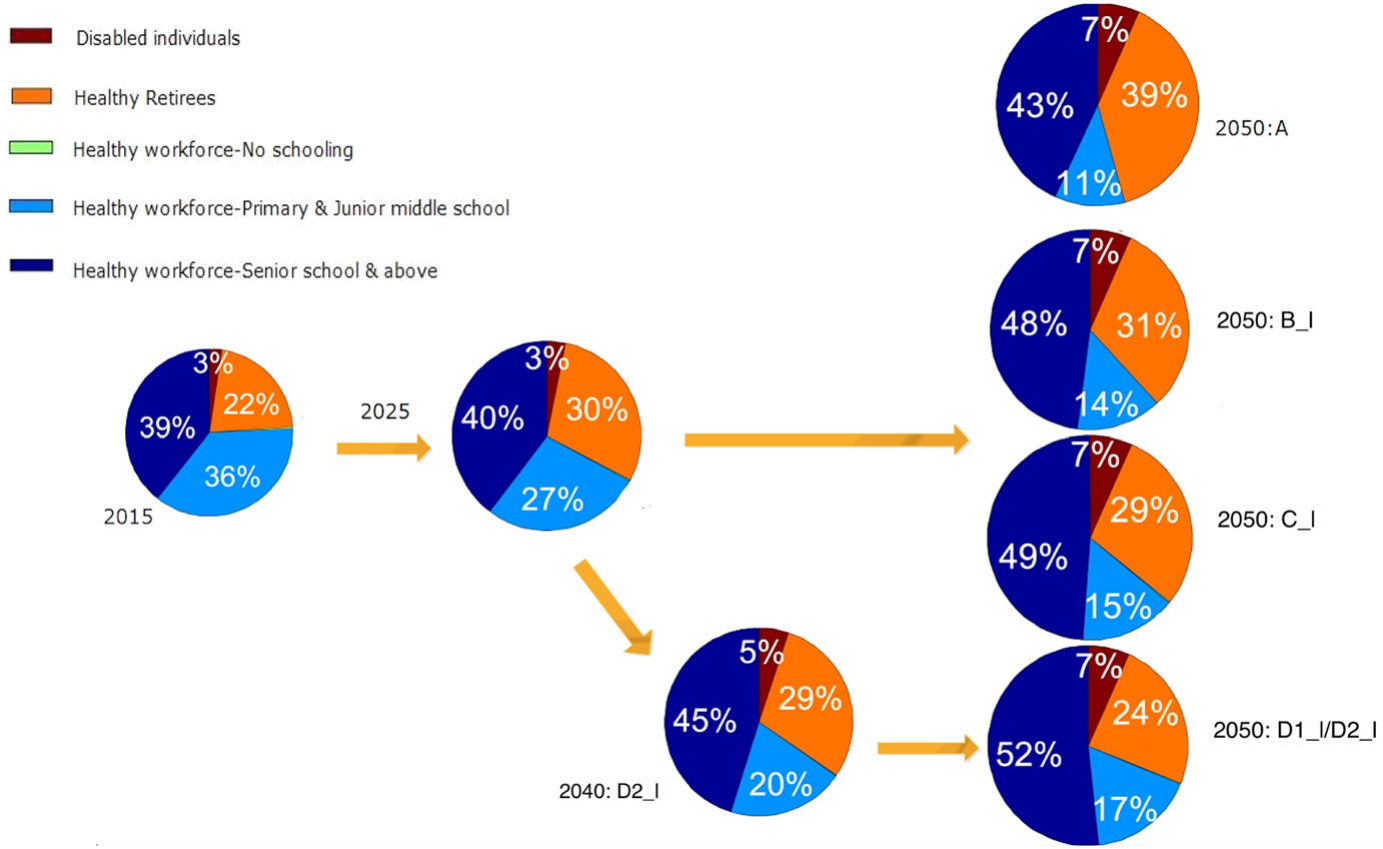
Working-age population in China by education and health under late retirement schemes adjusted from 2025 to 2050. The late retirement schemes include A (the current retirement ages remain unchanged) as the reference scheme, B_l (everyone will prolong retirement by 5 years from 2025 to 2050), C_l (females will retire at 60 and male at 65 from 2025 to 2050), D1_l (everyone will retire at 65 from 2025 to 2050), and D2_l (everyone will retire at 65 from 2025 to 2050 with females adjusted first). Note: An individual is categorized as “disabled” if he or she self-reported to be “unable to carry out regular work and daily activities,” or to be “not sure about their health status.” Those who reported “not sure” were less than 2% and the majority of them are older than age 70. The “healthy” category includes those who self-reported as “healthy” or as “basically can carry out regular work and daily activities”

Next, we incorporate the consideration for the quality of the workforce, measured by age-sex-specific education levels and health conditions. Figures 5 and 6 illustrate the scenarios of working-age population by different education and health status with an early and a late beginning time for adjustment. We see in Fig. 5 that if the retirement age remains unchanged, China’s high HC workforce (those who are healthy, with a high school and above education, and not retired) will gradually increase from 39% in 2015 to 43% in 2050. However, under Schemes B (*Add 5 Years*), C (*Females 60, Males 65*), and D (*65 for All*), this proportion will increase to 48, 49 and 52%, respectively, in 2050 if changes start to occur in 2015. In terms of the number of individuals, Schemes B, C and D will retain an additional 50.2 million, 59.4 million, and 88.1 million of high HC workforce, respectively, in 2050. There will also be an increase, though in a smaller magnitude, in the workforce with primary and junior middle school. The proportion of healthy retirees will reduce from 39% in *No Change s*cenario (scheme A) to 24% in the *65 for All* scenario (scheme D).

Figure 6 shows the different scenarios if changes start to occur in 2025. The smaller impact in the workforce composition in 2030 and 2040 can be seen when compared to Fig. 5. The gender-specific projections for the above scenarios (results not shown but available upon request) reveal that the gains in the high HC workforce due to retirement age adjustments are more substantial in females than males, mainly due to the rapid improvement of the female education in the last few decades which is expected to continue in the next few decades as discussed earlier. Among the additional high HC workforces retained by schemes C (*Females 60, Males 65*) and D (*65 for All*) in 2050, 53, and 68% are females, respectively.

### Cumulative Impact on Work Force Overtime

The previous calculations reveal gains in a particular year. Next, we show the cumulative person-year gains of workforce from 2010 in Tables 5 and 6. Scheme D1_e (*65 for All*, starting from 2015) yields the largest total cumulative gain from 2015 to 2050, namely 2376 million and 849 million person-years for females and males, respectively. This is because (1) it uses the oldest target age of retirement—65, (2) it starts in the earliest time point—2015, and (3) it adjusts the age for both male and female together from the very beginning. In contrast, Scheme B_l (*Add 5 Years,* starting from 2025) generates the smallest impact, amounting to about 474 million and 497 million person-years for females and for males, respectively, by 2050. Scheme D2_e, which only differs from D1_e in postponing the age for females to 60 first and then increase both gender to 65, has the second largest gain. It is also interesting to note that Scheme C_e (*Females 60, Males 65*, starting from 2015) and B_e (*Add 5 Years*, starting from 2015) have the third and fifth largest gains, respectively, suggesting that the beginning time might be a more important factor in affecting the cumulative gains than the target ages or gender-specific schedule in considering how to adjust the retirement age.

**Table 5:** Cumulative person-year gain of workforce in China under different retirement schemes in reference to Scheme A, 2010–2050 (Unit: thousand)

Nine retirement schemes	A	B_e	B_l	C_e	C_l	D1_e	D1_l	D2_e	D2_l
*Female*									
2010–2030	0	160,163	16,863	300,235	30,088	471,251	46,951	514,174	50,115
2010–2040	0	457,444	167,862	812,006	287,721	1,289,887	449,467	1,360,652	475,699
2010–2050	0	847,168	473,814	1,481,623	811,784	2,376,472	1,330,149	2,447,237	1,371,064
Average annual gain from 2010 to 2050	0	24,205	13,538	42,332	23,194	67,899	38,004	69,921	39,173
*Male*									
2010–2030	0	141,952	19,720	141,952	19,720	141,952	19,720	0	0
2010–2040	0	453,693	174,632	453,693	174,632	453,693	174,632	191,244	0
2010–2050	0	848,556	496,606	848,556	496,606	848,556	496,606	586,108	212,639
Average annual gain from 2010 to 2050	0	24,244	14,189	24,244	14,189	24,244	14,189	16,746	6,075
Total									
Average annual gain	0	48,449	27,726	66,577	37,383	92,144	52,193	86,667	45,249

The nine retirement schemes are A (the current retirement ages remain unchanged), B_e (everyone will prolong retirement by 5 years from 2015 to 2040), B_l (everyone will prolong retirement by 5 years from 2025 to 2050), C_e (females will retire at 60 and male at 65 from 2015 to 2040), C_l (females will retire at 60 and male at 65 from 2025 to 2050), D1_e (everyone will retire at 65 from 2015 to 2040), D1_l (everyone will retire at 65 from 2025 to 2050), D2_e (everyone will retire at 65 from 2015 to 2040 with females adjusted first), D2_l (everyone will retire at 65 from 2025 to 2050 with females adjusted first)

**Table 6:** Cumulative person-year gain of high human capital workforce in China under different retirement schemes in reference to Scheme A, 2010–2050 (Unit: thousand)

Nine retirement schemes	A	B_e	B_l	C_e	C_l	D1_e	D1_l	D2_e	D2_l
*Female*									
2010–2030	0	57,243	7489	91,292	11,416	146,698	18,906	150,824	19,026
2010–2040	0	207,716	84,849	306,001	123,478	487,913	202,744	494,136	201,572
2010–2050	0	454,593	281,688	651,166	399,812	1,051,708	674,707	1,057,931	670,734
Average annual gain from 2010 to 2050	0	12,988	8048	18,605	11,423	30,049	19,277	30,227	19,164
*Male*									
2010–2030	0	56,525	7374	56,525	7374	56,525	7374	0	0
2010–2040	0	193,135	76,450	193,135	76,450	193,135	76,450	88,269	0
2010–2050	0	410,230	255,269	410,230	255,269	410,230	255,269	305,364	121,406
Average annual gain from 2010 to 2050	0	11,721	7293	11,721	7293	11,721	7293	8725	3469
Total									
Average annual gain from 2010 to 2050	0	24,709	15,342	30,326	18,717	41,770	26,571	38,951	22,633

The nine retirement schemes are A (the current retirement ages remain unchanged), B_e (everyone will prolong retirement by 5 years from 2015 to 2040), B_l (everyone will prolong retirement by 5 years from 2025 to 2050), C_e (females will retire at 60 and male at 65 from 2015 to 2040), C_l (females will retire at 60 and male at 65 from 2025 to 2050), D1_e (everyone will retire at 65 from 2015 to 2040), D1_l (everyone will retire at 65 from 2025 to 2050), D2_e (everyone will retire at 65 from 2015 to 2040 with females adjusted first), D2_l (everyone will retire at 65 from 2025 to 2050 with females adjusted first)

Table 6 shows the gain in high human capital workforce specifically. The largest gain in the cumulative person-year gains in high HC workforce can be seen in the *65 for All* scenarios, particularly if changes happen earlier and to females first (scenario D2). The clear largest gain is among female high HC workers. For example, in Scheme D2_e (*Female First, 65 for All*, starting from 2015), 1058 million person-years of female high HC workforce will be added over the 25 years, amounting to an annual average of 30,227 female high HC workers. In contrast, under Scheme B_l (*Add 5 Years*, starting from 2025), the corresponding gain will only be 282 million cumulative person-years by 2050 and an annual average of 8048 workers.

### Worker/Retiree Ratio

Another important indicator to evaluate the impact of various scenarios is to examine the size of the workforce relative to retirees. We examine the changes of the worker/retiree ratios, calculated as the ratio of workforce population per retiree, under different schemes. Additionally, in order to better show the impact of retirement reform on the quality of labor force, we similarly create a high human capital workforce/retiree ratio (short for high HC worker/retiree ratio), calculated as the total number of the high HC workers (those who are healthy, not retired, and with a high school and above education) divided by the total number of retirees.

**Fig. 7:**
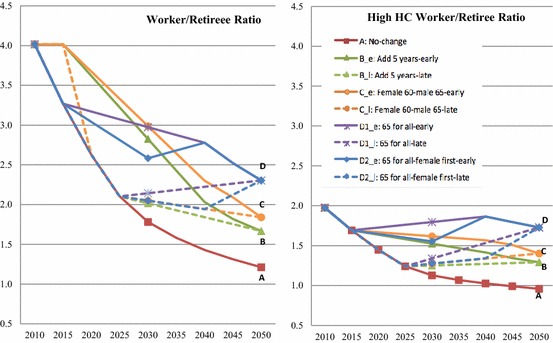
Worker/retiree ratio under nine retirement schemes in China, 2010 to 2050. The nine retirement schemes are A (the current retirement ages remain unchanged), B_e (everyone will prolong retirement by 5 years from 2015 to 2040), B_l (everyone will prolong retirement by 5 years from 2025 to 2050), C_e (females will retire at 60 and male at 65 from 2015 to 2040), C_l (females will retire at 60 and male at 65 from 2025 to 2050), D1_e (everyone will retire at 65 from 2015 to 2040), D1_l (everyone will retire at 65 from 2025 to 2050), D2_e (everyone will retire at 65 from 2015 to 2040 with females adjusted first), D2_l (everyone will retire at 65 from 2025 to 2050 with females adjusted first). The worker/retiree ratio is calculated as the total number of the workforce divided by the total number of retirees. The high HC worker/retiree ratio (the high human capital worker/retiree ratio) is calculated as the total number of the high human capital workforce (those who are healthy, not retired, and with a high school and above education) divided by the total number of retirees

**Fig. 8:**
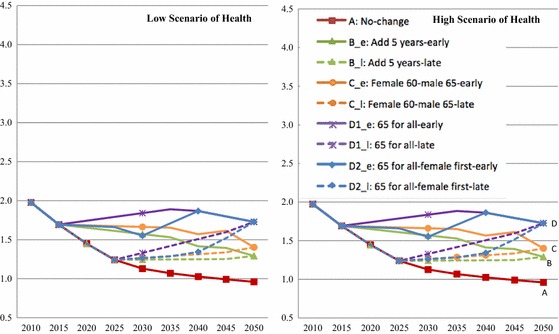
High HC workforce/retiree ratios of under the nine retirement schemes by low and high scenarios of health. The high HC worker/retiree ratio (the high human capital workforce/retiree ratio) is calculated as the total number of the high human capital workforce (those who are healthy, not retired, and with a high school and above education) divided by the total number of retirees. For the low scenario of health, we assume the proportion of unhealthy Chinese elderly (50 +) will decrease by 10% from 2005 to 2050. For the high scenario of health, we assume the proportion of unhealthy Chinese elderly (50 +) will gradually increase by 10% from 2005 to 2050. The nine retirement schemes are A (the current retirement ages remain unchanged), B_e (everyone will prolong retirement by 5 years from 2015 to 2040), B_l (everyone will prolong retirement by 5 years from 2025 to 2050), C_e (females will retire at 60 and male at 65 from 2015 to 2040), C_l (females will retire at 60 and male at 65 from 2025 to 2050), D1_e (everyone will retire at 65 from 2015 to 2040), D1_l (everyone will retire at 65 from 2025 to 2050), D2_e (everyone will retire at 65 from 2015 to 2040 with females adjusted first), D2_l (everyone will retire at 65 from 2025 to 2050 with females adjusted first)

As shown in Fig. 7, if the retirement age remains unchanged (scheme A), the worker/retiree ratio will decline drastically from about 4.0 in 2010 to 1.2 in 2050, which means there will be 1.2 working-age persons per retiree in 2050. Under such a scenario, the working population’s burden is very high, particularly considering the fact that most working-age population also need to support young dependents. Adjusting retirement age will decelerate the increase in workforce/retiree ratios by varying degrees under different scenarios. If Scheme D (*65 for All*) is adopted, the support ratio will increase to about 2.3 by 2050 as opposed to 1.2, which means every retiree has approximately one additional working-age person to support him or her. The impact is even stronger if we use the high HC worker/retiree ratio in the calculation. If the retirement ages remain unchanged (scheme A), such a ratio will almost be reduced by half (from 2 to 1) by 2050, but if Scheme D (*65 for All*) is implemented, this ratio will only slightly drop from 2.0 to about 1.7. The effects of Schemes B (*Add 5 Years*) and C (*Females 60, Males 65*) fall in between Schemes A and D (shown in Fig. 7). Under Schemes B and C, the worker/retiree ratios will be 1.7 and 1.8, respectively, by 2050, and the high HC worker/retiree ratios will be 1.3 and 1.4.

## Conclusion and discussion

Whether and how the retirement age should be changed is a complex issue currently under debate in China, a nation that has a rapidly aging and the largest elderly population in the world. This study echoes previous proposals that advocated the adjustment of retirement age for the solvency of the nation’s pension account, but links such initiatives to a different but equally important issue—the supply of human capital and the implications for future economic development in China. We review major policy proposals, develop alternative schemes for adjusting the retirement age, and project labor force compositions under each scheme for the next four decades. The ProFamy method is applied to the most recent Chinese micro-level census data in the forecasts. To our knowledge, this is the first systematic empirical study that examines how adjustment of retirement age could affect both the size and quality of labor force in China. Unique to the projections is the incorporation of education and health, the two major components of human capital, to reflect the changes of quality of labor force in China over time, which is pivotal for China’s transformation from a cheap-labor-intensive model to an innovative technologically oriented model in development.

The projections in this study capture the major social-demographic impetuses shaping China’s future labor force, including the relaxing of one-child policy, population aging, rapid urbanization, and education expansion. By modeling these factors together in the projections, we reveal the basic dynamics of the future labor force in China, and capture how different schemes of retirement ages shape the trajectories. This study provides solid forecasts based on well-researched assumptions to show the effects and pathways under various policy proposals for adjusting the retirement ages, which are useful not only for policymakers in China but also have implications for other countries contemplating similar changes in the retirement policy.

Moreover, the projections in this study use health and education to elaborate on the impact of retirement reforms on the structure of human capital in China, which provides richer insights than projections only based on chronical age. Granted, more refined indicators of human capital, such as cognitive test scores, emotional quotients, or psychological traits, can better capture an individual’s human capital. Unfortunately, these measures are not available in the census data. Future work can further refine this line of research. Skirbekk et al. (2012), for example, recently added cognitive functioning measure to refine the traditional age-based measure of dependency ratio. Using such measures, they show that China has a more prominent challenge of population aging than the Northern European countries, though the proportion of elderly is relatively lower in China. Projections in this study are consistent with this line of method progression. We also believe the relatively worse cognitive functioning of the Chinese elderly may affect the size and quality of human capital in China, and warrants further investigations (Chan et al. 2013; Wu et al. 2014).

The different demographic scenarios projected in this study show that delaying age of retirement will significantly increase the workforce size, and most importantly, improve the quality of the workforce and reduce the worker/retiree ratios in China. The effectiveness of adjusting retirement age is particularly evident when improvement in education over time is taken into consideration. That is, postponing the retirement age will not only retain additional individuals who will otherwise retire at a relatively early age while still healthy, but also retain individuals with increasingly higher human capital over time in the labor force. These patterns suggest that it is high time to reap the benefits of China’s investment in human resources in the last few decades, especially the investment in women’s education. Because the cohorts that have benefitted from the education expansion since 1999 (resulting in sevenfolds’ increase in annual college enrollment by 2015) will start to enter retirement age around 2040, the revised retirement age will thus retain significantly a larger number of higher human capital workers for China by then. It is crucial to note the large gap in gains by gender. As female college enrollment has surpassed that for males since 2009 (Yeung 2013), increasing age of retirement for females will generate a significantly larger benefit to the workforce when this cohorts of females start to enter retirement age.

We show the relative impact on the labor force of alternative scenarios proposed. According to the forecasted scenarios, Scheme D1_e (*65 for All*, starting from 2015) will produce the largest gain in number of workers and is most effective with regard to reaping the benefit of the nation’s human capital investment. However, it may also face the highest resistance from the public, particularly from females as the magnitude of adjustment is largest for them, particularly for the female workers (adjust from 50 to 65, starting from 2015).

Short of this most drastic scheme, policymakers could follow two pathways in considering other options of adjustment. The first pathway could prioritize the target ages, namely, starting from the oldest target age and then consider lowering the age of retirement if resistance from public is too high. Along this line, Scheme D2_e, (*Female First*, *65 for All*, starting from 2015) could be tested as the first alternative to Scheme D1_e (*65 for All*, starting from 2015). This scheme can be expected to face less resistance from both males and females as males will not be affected in the first 15 years under this scheme. Likewise, Scheme D1_l (*65 for all*, starting from 2025) could be considered next as it delays the same adjustments for 10 years so that all current retirees-to-be will not be affected. However, D1_l scheme has a much weaker impact compared to D2_e or D1_e. Scheme D2_l (*females first, 65 for all*, starting from 2025) is expected to face an even lower resistance though the impact will clearly also be much smaller. Schemes C (*Females 60, Males 65*) and B (*Add 5 Years*) both involve even smaller and more gradual adjustments.

Another line of policy alternatives could center upon the beginning time of the retirement age adjustment, which we have shown to have a substantial influence on the cumulative gains of labor force. If Scheme D1_e is not politically viable, Schemes D2_e, C_e and B_e, all starting from 2015, could be considered consecutively. Starting the adjustments in 2025 produces a much smaller impact on the labor market.

Based on 2005 census data, only about 1.2% of females were not fit to work by the age of 54, and about one-third of them had an education of high school and above, and for males, only about 2.0% were not fit to work by age 60 and about 30% of them had a high school and above education. Given the increasing living expenditure in China, Scheme C_e (*Females 60, Males 65,* starting from 2015) is an option that may achieve a reasonable balance between the gain in the work force and resistance from the public. Under this scheme, a cumulative of 1.48 billion female and 849 million male person-years, respectively, working out to be an average of an annual gain of 42 million females and 24 million males per year, will be added to the workforce, respectively, by 2050. Of these cumulatively added workforce, 651 million female and 410 million male person-years will be of high human capital, which work out to be 18.6 million female and 11.7 million male workers per year. The worker/retiree ratio will increase by 52%, with the high human capital worker/retiree ratio increases by 46% in 2050 compare to the *No Change* scenario (scheme A). These are highly significant impact.

Results presented here should be interpreted with caution. First, although the assumptions made in the projections are highly plausible, uncertainties remain. Summary parameters used in the ProFamy projections are all based on evidence in well-established literature about the Chinese demographic trends and thus represent plausible trajectories. We have also tested projections under different sets of assumptions (available online for these supplementary materials) and find that the main patterns as projected above are not substantially different, though the relative magnitude of the impact varies. Nevertheless, it is worth emphasizing that forecasts of this study are national projections, and the substantial regional disparity of China in population aging, public health and education, and economic development may lead to significantly different local trajectories of human capital. That is, results of this paper are not meant to be applied to region-specific trajectories.

Next, the indictors of human capital could be more refined to go beyond education and disability. Skills and test scores have recently been used by international surveys such as PISA and PIAAC, but unfortunately, none of these measured are available in the Chinese data. Moreover, our projections consider human capital gains only from the supply perspective. Critics of postponing retirement age worry about youth employment as they argue prolonged stay of mature workers in the job market may affect the opportunities of youth. However, evidence based on panel data in 22 OECD countries (Kalwij et al. 2009) showed that postponing retirement age had no adverse effect on youth employment from the 1960s to 2000s because the hypothesis that employment of the young and old are substitutes is invalid. Moreover, based on data from 91 countries and regions, Cai (2009) also found no evidence that delaying the age of retirement is associated with unemployment rates. Furthermore, our proposed scenarios start in either 2015 or 2025, when newly added labor force will generally be in decline in China, thus partially easing the pressure of unemployment for young workers (as shown in Fig. 1).

Finally, we are well aware of the difference between the policy-imposed official retirement age and the actual retirement age. As shown in Table 1, individuals tend to retire earlier than the policy-designated age in the West, and the situation is similar in China: although there is no consensus about the average of the actual retirement age, scholars have estimated it to be around 55 for males and 50 for females in the recent decade (Cai 2009; MHRSS 2013). To account for this issue, we also project a scenario in which the estimated actual retirement ages are prolonged by 5 years from 2015 to 2040 for both men and women (results not shown but available upon request). As the target retirement ages are younger than the ones we used for the official retirement ages, the gains in human capital in these scenarios are lower than the levels shown in the projection, but the general patterns remain. The projections based on actual retirement age could reflect the reality better.

This study has implications for the current pension reform of China. Our projections not only illustrate the potential impact of prolonged retirement ages in improving solvency of the national pension account, but also provide support for the recent proposal to establish “the third pillar” in the current pension reform of China (Dong and Yao 2017), which refers to private-based funding in addition to public and employer-based funding as the first and second pillars, respectively (World Economic Forum 2017). Advocates for the third pillar argue for a multilayered scheme to allow for more flexibility and sustainability, particularly for those young, educated, and with middle/high income, who have shown keen interests for the individualized scheme (Dong and Yao 2017). According to our projections, if retirement age is postponed, more of these individuals will be retained in the future labor force, and the preference and demand for private pension may increase, which could be a possible scenario for the Chinese pension system in the future. It is not, however, the aim of this study to address the causal effects of policy interventions on labor market or retirement behavior as some recent works have done (e.g., Arpaia et al. 2011; Geyer et al. 2016).

Apart from the added number and quality of the workforce, another potential gain for postponing the retirement age is that a later exit from the labor force could help the elderly to maintain the cognitive functioning, because cognitively challenging activities such as those at work could enhance the aging brain’s neuroplasticity (Bonsang et al. 2012; Park and Bischof 2013). Such benefit reminds us that prolonging the retirement age should aim to benefit the entire society for a more sustainable future rather than only as a temporary solution for a financial challenge. The notions of active, productive, or successful aging have started to spread from Europe and America to Asia as a new orientation for promoting a better aging society, encouraging a physically healthy, economically productive and socially active later life, both at the individual and society level (Walker and Maltby 2012; Rowe and Kahn 2015). These new policy perspectives should be considered along with the proposals of postponing the age of retirement in China.

It can be expected that implementing reforms in retirement age will encounter great difficulties. Although state plays a dominate role in policy decisions in China, without a holistic consideration of the needs of the older adults and the prevailing social norms, the retirement reform in China is likely to fail. To help older adults maintain their economic activities in later ages, it is imperative to ensure employment opportunities, flexible work arraignments and renumeration, age-friendly work environment, and training opportunities. For a smooth and effective reform to postpone the retirement age, it is also necessary to understand and reconcile interests and concerns of different stakeholders in the labor market. The decision to retire or to continue to work are often complex results of factors including replacement rate of pension, personal life events, savings, work condition, health, and family circumstances. Among employers, on the other hand, there are still practices of ageism to devalue the productivity of elderly employee and to lay off elderly employees for higher payroll and health insurance. A good retirement reform thus needs a comprehensive policy package effectively addressing these issues rather than only postponing the retirement age.

In China, a successful retirement reform should prioritize the needs of vulnerable subpopulations in the labor market. There should be programs to protect the rights of aged workers, especially those in labor-intensive sectors. Moreover, the retirement reform should not aggravate the extant pension inequality in China, which has already been substantial across the public and non-public sectors. In particular, the working class females deserve special considerations who may face a more drastic adjustment due to their relatively early retirement age currently (i.e., age 50) and their more disadvantaged socioeconomic status than males.

Delaying retirement age may also encounter backlash as it challenges the social stereotype of aged adults in China. Age 60 has traditionally been acknowledged as the marker of being old in China, which has been reinforced by the mandatory retirement age policy. According to a recent report, the perceived age marker of being old in China is 63.70 for men and 59.95 for women (Liang 2014). Under such perceptions, there exists strong ageism against elderly to work: Employees are not expected to seek new jobs after retirement; the age specification against older job seekers is a common practice in the current labor market; and the training and educational programs for seniors are close to non-existing (Lu 2009; Boshier 2012). Lou et al. (2013) even suggest that Chinese college students currently held more negative attitudes toward the elderly than their American peers, even though the traditional Confucian culture emphasizes respects to the elderly. To overcome these barriers, a campaign against ageism seems necessary in China when considering postponing retirement age as an important developmental policy solution.

### Electronic supplementary material

Below is the link to the electronic supplementary material.

Supplementary material 1 (PDF 357 kb)

## Appendix

See Tables 7, 8, 9 and Fig. 8.

**Table 7:** Major demographic parameters of China from 2010 to 2050 assumed in the projections of ProFamy

Year	Life expectancy at birth		Mean age at first marriage		Mean age at birth	Total fertility rate	General marriage rate	General divorce rate	Proportion in total population
	Women	Men	Women	Men					
*Urban*									
2010	77.87	74.21	25.34	27.1	29.72	1.24	0.0899	0.0082	0.50
2015	78.96	75.22	26.00	27.97	30.81	1.67	0.0921	0.0085	0.55
2020	80.36	76.44	26.66	28.84	31.89	1.67	0.0944	0.0088	0.63
2030	81.76	77.65	27.48	30.13	33.05	1.67	0.0989	0.0094	0.71
2035	82.50	78.36	27.91	30.80	33.65	1.72	0.0989	0.0094	0.75
2050	83.99	80.54	28.33	31.47	34.25	1.72	0.0989	0.0094	0.87
*Rural*									
2010	73.88	70.07	23.64	25.64	27.89	2.01	0.0853	0.0032	0.50
2015	74.79	71.09	24.52	26.52	28.62	2.15	0.0879	0.0033	0.45
2020	76.21	72.35	25.4	27.4	29.35	2.16	0.0904	0.0034	0.37
2030	77.64	73.61	26.49	28.62	30.12	2.16	0.0956	0.0037	0.29
2035	78.42	76.40	27.06	29.26	30.52	2.21	0.0956	0.0037	0.25
2050	80.72	76.50	27.62	29.89	30.91	2.13	0.0956	0.0037	0.13

**Table 8:** Assumption on gender–age-specific prevalence of unhealthy condition from 2010 to 2050 in urban China (%). *Source*: Based on the National 1% Population Survey of China in 2005

Age group	Low scenario		Medium scenario		High scenario	
	Males	Females	Males	Females	Males	Females
0–4	0.30	0.27	0.30	0.27	0.30	0.27
5–9	0.26	0.19	0.26	0.19	0.26	0.19
10–14	0.31	0.23	0.31	0.23	0.31	0.23
15–19	0.39	0.29	0.39	0.29	0.39	0.29
20–24	0.45	0.32	0.45	0.32	0.45	0.32
25–29	0.45	0.33	0.45	0.33	0.45	0.33
30–34	0.55	0.40	0.55	0.40	0.55	0.40
35–39	0.64	0.49	0.64	0.49	0.64	0.49
40–44	0.89	0.65	0.89	0.65	0.89	0.65
45–49	1.18	0.89	1.18	0.89	1.18	0.89
50–54	1.40	1.09	1.56	1.21	1.72	1.33
55–59	1.83	1.85	2.03	2.06	2.23	2.27
60–64	3.06	3.89	3.40	4.32	3.74	4.75
65–69	5.48	6.94	6.09	7.71	6.70	8.48
70–74	10.31	11.69	11.46	12.99	12.61	14.29
75–79	14.59	18.77	16.21	20.85	17.83	22.94
80–84	21.96	27.60	24.40	30.67	26.84	33.74
85–89	29.17	35.76	32.41	39.73	35.65	43.70
90–94	42.80	41.48	47.55	46.09	52.31	50.70
95–99	47.87	52.45	53.19	58.28	58.51	64.11
100+	51.43	48.47	57.14	53.85	62.85	59.24

An individual is categorized as “disabled”“ if he or she self-reported to be “unable to carry out regular work and daily activities,” or to be “not sure about their health status.” Those who reported “not sure” were less than 2% and the majority of them are older than age 70. The “healthy” category includes those who self-reported as “healthy” or as “basically can carry out regular work and daily activities.” For the low scenario, we assume the proportion of unhealthy Chinese elderly (50 +) will decrease by 10% from 2005 to 2050. For the medium scenario, we assume the proportion of unhealthy Chinese elderly (50 +) will not change from 2005 to 2050. For the high scenario, we assume the proportion of unhealthy Chinese elderly (50 +) will gradually increase by 10% from 2005 to 2050

**Table 9:** Assumption on gender–age-specific prevalence of education levels from 2010 to 2050 in urban China (%). *Source*: The gender–age-specific prevalence of education levels in 2010 is based on the 1% sample from the 2010 China Census

Age group	No schooling				Primary school				Junior middle school				Senior middle school				College and above			
	Males		Females		Males		Females		Males		Females		Males		Females		Males		Females	
	2010	2050	2010	2050	2010	2050	2010	2050	2010	2050	2010	2050	2010	2050	2010	2050	2010	2050	2010	2050
0–4	100	100	100	100	0	0	0	0	0	0	0	0	0	0	0	0	0	0	0	0
5–9	24.7	24.7	24.5	24.5	75.0	75.0	75.1	75.1	0.3	0.3	0.4	0.4	0.0	0.0	0.0	0.0	0.0	0.0	0.0	0.0
10–14	0.3	0.3	0.2	0.2	52.2	52.2	51.4	51.4	46.0	46.0	46.7	46.7	1.6	1.6	1.1	0.9	0.0	0.0	0.0	0.0
15–19	0.1	0.0	0.1	0	1.2	0.0	1.3	0.0	32.7	40.0	29.6	40.0	54.1	54.0	55.1	54.0	11.8	6.0	7.9	6.0
20–24	0.2	0.0	0.2	0	2.4	0.0	2.7	0.0	33.3	10.0	33.8	10.0	24.5	32.0	22.5	32.0	39.6	58.0	40.7	58.0
25–29	0.2	0.0	0.4	0	3.0	0.0	4.2	0.0	40.0	10.0	40.5	10.0	23.9	32.0	21.4	32.0	32.9	58.0	33.5	58.0
30–34	0.3	0.0	0.5	0.0	5.2	0.4	8.3	0.5	42.6	13.7	43.0	13.8	24.4	29.2	22.2	28.9	27.4	56.6	26.0	56.7
35–39	0.3	0.1	2.6	0.9	8.5	0.8	19	1.0	46.9	17.5	46.8	17.6	22.7	28.5	18.6	27.9	21.5	53.2	13.0	53.4
40–44	0.5	0.1	3.5	0.1	11.4	1.1	16.9	1.6	49.4	21.2	41.6	21.5	21.1	27.7	27.4	26.8	17.6	49.8	10.5	50.1
45–49	0.6	0.1	6.7	0.2	10.7	1.5	22.9	2.1	45.3	25.0	34.5	25.3	26.3	26.9	28.8	25.7	17.1	46.5	7.0	46.8
50–54	1.1	0.1	4.5	0.2	16.2	1.9	26.2	2.6	41.0	28.7	36.7	29.1	29.0	26.2	25.8	24.6	12.6	43.1	6.8	43.5
55–59	2.2	0.2	8.0	0.3	26.4	2.3	37.0	3.1	43.1	32.5	36.2	32.9	17.7	25.4	12.9	23.6	10.4	39.7	5.8	40.2
60–64	3.1	0.2	11.4	0.3	35.9	2.6	45.8	3.6	36.1	36.2	28.3	36.7	14.8	24.6	10.2	22.5	10.0	36.3	4.3	36.8
65–69	4.8	0.2	17.6	0.4	36.9	3.0	44.8	4.2	31.8	40.0	22.5	40.5	15.2	23.9	10.3	21.4	11.3	32.9	4.8	33.5
70–74	8.4	0.3	27.5	0.5	40.6	5.2	43.9	8.3	24.4	42.6	15.7	43.0	14.0	24.4	8.0	22.2	12.6	27.4	4.9	26.0
75–79	12.4	0.3	41.3	0.8	44.0	8.5	42.0	12.6	22.2	46.9	9.3	48.9	11.4	22.7	4.5	20.0	10.0	21.5	2.9	17.7
80–84	18.6	0.5	54.1	1.3	44.8	11.4	34.8	18.0	19.2	49.4	6.8	49.8	10.2	21.1	2.5	18.2	7.2	17.6	1.8	12.7
85–89	21.3	0.6	62.3	2.0	50.3	10.7	29.7	17.8	14.2	45.3	4.3	45.5	7.8	26.3	2.3	24.0	6.4	17.2	1.4	10.7
90–94	39.0	1.1	69.9	4.5	39.0	16.2	25.3	26.2	13.2	41.0	3.0	36.7	3.4	29.0	0.8	25.8	5.4	12.6	1.0	6.8
95–99	28.6	2.2	75.3	8.0	34.7	26.4	17.6	37.0	26.5	43.1	2.4	36.2	10.2	17.7	1.2	12.9	0.0	10.4	3.5	5.8
100+	0.0	3.1	69.2	11.4	50.0	35.9	15.4	45.8	0.0	36.1	0.0	28.3	50.0	14.8	15.4	10.2	0.0	10.0	0.0	4.3

## Footnotes

1 We also try the starting year of 2016 and 2020 and get similar results. Due to the space constraints, these results are not presented in the manuscript.

2 The colored version of all figures is available upon request.
