# Supplementary material for: Age of Retirement and Human Capital in an Aging China, 2015–2050
Source: Eur J Popul. 2018 Feb 13;35(1):29–62. doi: 10.1007/s10680-018-9467-3 (PMC6357252; doi:10.1007/s10680-018-9467-3)
Supplement: Supplementary file 1 — Supplementary material 1 (PDF 357 kb) [file 10680_2018_9467_MOESM1_ESM.pdf]

Online Supplementary Material

In addition to the projections presented in the main text, we further examine the effect of retirement age adjustments on the future labor force of China under different assumptions of fertility and mortality, two major parameters affecting the population size and structure. We develop two additional sets of assumptions, the low assumptions and the high assumptions, whereas the assumptions used for the projections in the main text are thereafter named as the medium assumptions. For the low assumptions, the fertility rates are set higher and the life expectancies are set lower than the medium assumptions; in contrast, the fertility rates are set lower and the life expectancies are set higher in the high assumptions than the medium assumptions. With higher fertility and lower life expectancy, the low assumptions correspond to a scenario with a relatively smaller scale and slower speed of population aging, and meanwhile the high assumption represents the other end of the spectrum. Details of these assumed parameters are summarized as blow:

		**Low Assumptions**		**Medium Assumptions**		**High Assumptions**	
	2010	2030	2050	2030	2050	2030	2050
**Urban**							
Total fertility rate	1.24	1.96	2.12	1.67	1.72	1.24	1.24
Male life expectancy	74.21	75.71	78.53	77.65	80.54	79.60	82.56
Female life expectancy	77.87	79.72	81.89	81.76	83.99	83.81	86.09
**Rural**							
Total fertility rate	2.01	2.35	2.29	2.16	2.13	2.03	1.94
Male life expectancy	70.07	71.77	74.59	73.61	76.50	75.45	78.42
Female life expectancy	73.88	75.69	78.70	77.64	80.72	79.58	82.73

Assumptions	**Low Assumptions**		**Medium Assumptions**		**High Assumptions**	
	Workforce	Retiree	Workforce	Retiree	Workforce	Retiree
Population in 2010	460,967,916	114,727,989	460,967,916	114,727,989	460,967,916	114,727,989
**Change in 2050**						
A	31%	277%	22%	304%	14%	331%
B_e	48%	210%	39%	235%	31%	261%
B_l	48%	210%	39%	235%	31%	261%
C_e	53%	189%	44%	214%	37%	240%
C_l	53%	189%	44%	214%	37%	240%
D1_e	64%	146%	55%	170%	48%	195%
D1_l	64%	146%	55%	170%	48%	195%
D2_e	64%	146%	55%	170%	48%	195%
D2_l	64%	146%	55%	170%	48%	195%

Note: The nine retirement schemes are A (the current retirement ages remain unchanged), B_e (everyone will prolong retirement by 5 years from 2015 to 2040), B_l (everyone will prolong retirement by 5 years from 2025 to 2050), C_e (females will retire at 60 and male at 65 from 2015 to 2040), C_l (females will retire at 60 and male at 65 from 2025 to 2050), D1_e (everyone will retire at 65 from 2015 to 2040), D1_l (everyone will retire at 65 from 2025 to 2050), D2_e (everyone will retire at 65 from 2015 to 2040 with females adjusted first), D2_l (everyone will retire at 65 from 2025 to 2050 with females adjusted first)

The table above reports the relative change in the size of labor force from 2010 to 2050 under the three sets of assumptions. These results reveal that the low and high assumptions generate a similar pattern of changes with the medium assuptions across the nine retirement schemes, though the scale and speed of population aging are different.

The following two figures further present the worker/retiree ratio (the total number of work force divided by total number of retirees) of the nine retirement schemes, respectively for the low and high assumptions. As can been seen, the pattern of changes is similar to Figure 5 in the main text under the medium assumptions, though the low assumptions produce higher worker/retiree ratios, whereas the high assumptions have lower worker/retiree ratios.

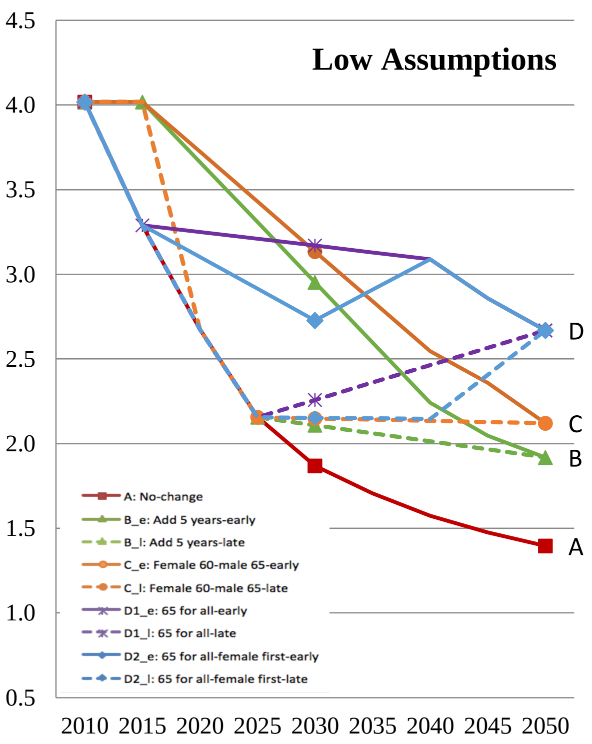

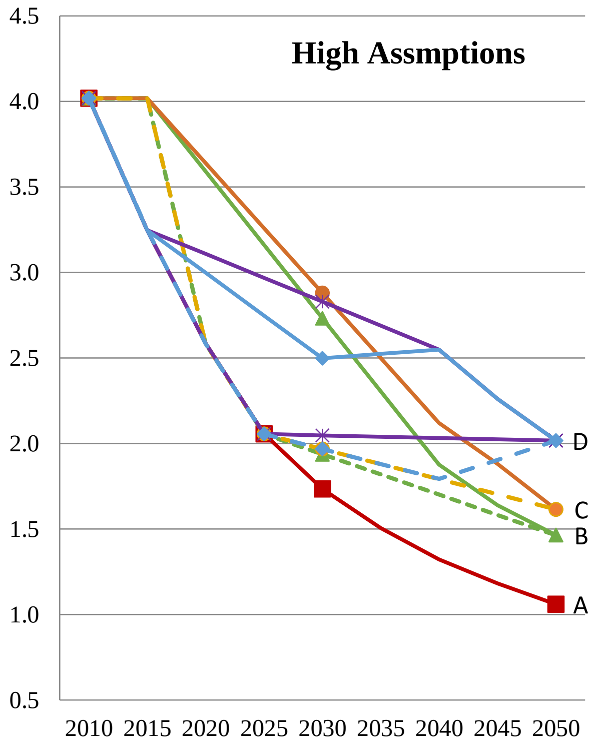

Note: The nine retirement schemes are A (the current retirement ages remain unchanged), B_e (everyone will prolong retirement by 5 years from 2015 to 2040), B_l (everyone will prolong retirement by 5 years from 2025 to 2050), C_e (females will retire at 60 and male at 65 from 2015 to 2040), C_l (females will retire at 60 and male at 65 from 2025 to 2050), D1_e (everyone will retire at 65 from 2015 to 2040), D1_l (everyone will retire at 65 from 2025 to 2050), D2_e (everyone will retire at 65 from 2015 to 2040 with females adjusted first), D2_l (everyone will retire at 65 from 2025 to 2050 with females adjusted first)
